# A study on the dynamic differences and component correlations of astringency in green tea, black tea and oolong tea based on TI/TDS and LC-MS

**DOI:** 10.1016/j.fochx.2026.104130

**Published:** 2026-06-24

**Authors:** Wei Mao, Yajuan Li, Fangfang Yan, Hui Zhou, Xiankang Fan, Zhonghua Liu, Maiquan Li, Jie Luo

**Affiliations:** a College of Food Science and Technology, Hunan Agricultural University, Changsha 410114, China; b College of Horticulture, Hunan Agricultural University, 410128 Changsha, China

**Keywords:** Tea, Astringency, Sub-quality, Time intensity, Metabolomics

## Abstract

The dynamic patterns and chemical drivers of astringency across tea varieties remain underexplored. This study integrated time-intensity (TI), temporal dominance of sensations (TDS), electronic tongue, and LC-MS analyses to evaluate astringency in six green, black, and oolong teas. TI results indicated that green tea exhibited higher peak intensity, larger area under the curve (AUC), longer duration, and faster onset compared to oolong and black teas. TDS analysis revealed dominant sensations: “harsh” and “coarse grain” for green tea, “smooth” and “dry” for black tea, and alternating “rough” and “dry” for oolong tea. LC-MS profiling linked high astringency to anthocyanins, quercetin derivatives, and catechin oxidation products. Roughness correlated with polymerized catechins, granularity with flavanols, and dryness with oxidized polyphenols. Overall, polyphenol type and structure are key determinants of astringency intensity and sub-quality variations among tea types.

## 1 Introduction

Tea is a globally popular beverage renowned for its unique flavor, which significantly influences consumer preferences. Among its diverse flavor dimensions, astringency plays a pivotal role as a core sensory attribute and a key indicator of consumer acceptance (Jian-Hui et al., 2022; Zhang et al., 2020). Astringency arises from interactions between polyphenol compounds and salivary proteins, leading to reduced lubrication of the oral epithelium (Bajec & Pickering, 2008). It can be defined as “a sensory complex involving epithelial contraction, tightening, or puckering due to contact with substances such as alum or tannins” (L. Chen et al., 2021b; Robichaud & Noble, 1990). This sensation is not a static experience but a dynamic process that evolves over time in the mouth(L. Vidal et al., 2017).

However, research on tea astringency has largely relied on static assessments. Most conventional methods involve static sensory scoring or single-time-point chemical analyses (Deng et al., 2022; Drobna et al., 2004; X. Wang et al., 2022). While these approaches provide foundational data, they fail to capture the temporal evolution of astringency in the oral cavity. Astringency encompasses onset, development, peak, and fading—quintessential time-dependent phenomena. Traditional descriptive analysis identifies sensory attributes but lacks resolution for their dynamics. In contrast, time-intensity (TI) analysis accurately depicts the continuous change in a specific attribute (e.g., astringency intensity) over time, yielding kinetic parameters such as onset time, rate of increase, peak intensity, time to peak, and decay rate (Gotow et al., 2015; Schmitt et al., 1984; Wang et al., 2022); Similarly, temporal dominance of sensations (TDS) tracks which sensory attribute dominates perception at any moment during tasting, elucidating dynamic interactions between astringency and other flavor attributes (Le Révérend et al., 2008; Rizo et al., 2019; Rodrigues et al., 2017). Notably, astringency is not monolithic; it comprises sub-qualities such as harsh, coarse grain, dry, and rough (Brossard et al., 2021; Wang et al., 2024). These sub-qualities likely vary across tea types due to differences in chemical composition(François et al., 2006; Vidal et al., 2018).

While TI and TDS methodologies have been successfully employed to explore the dynamic characteristics of astringency in beverages like beer and wine (Zhao et al., 2019). their application in tea research, particularly for a systematic investigation of astringency sub-qualities alongside chemical profiling, remains limited. The chemical basis for these sensory differences is rooted in tea processing. For example, in green tea processing, enzyme activity is rapidly inhibited, preserving a high content of simple catechins (Xu et al., 2018); In contrast, black tea undergoes full enzymatic oxidation, leading to the formation of theaflavins and thearubigins, which are associated with a milder, rounder astringency (Chen et al., 2021). Oolong tea, being semi-fermented, occupies a middle ground, with its specific processing (partial fermentation and roasting) yielding a complex and diverse polyphenol profile (Hao et al., 2024). These fundamental chemical differences are anticipated to directly govern the significant variations in astringency intensity, onset rate, duration, and dynamic patterns across different tea types during consumption.

Thus, this study selected six representative commercial teas from the three major types (green, oolong, and black). We employed a multimodal approach: TI and TDS for temporal dynamics of astringency intensity and sub-qualities; electronic tongue for objective intensity measurement to validate sensory data; and LC-MS for high-throughput chemical profiling. By integrating these datasets, we elucidate relationships between polyphenol composition/structure and astringency dimensions (intensity, temporal parameters, sub-qualities). The goal is to advance understanding of the molecular mechanisms underlying astringency formation and dynamic expression in tea.

## 2 Materials and methods

### 2.1 Materials

#### 2.1.1 Sample

This study selected six representative types of commercially available tea, green tea: Maojian green tea (Henan, with a pan-frying temperature of 220 °C), Longjing green tea (Zhejiang, with a pan-frying temperature of 200 °C), and oolong tea: Tie guan yin (Fujian, with a fermentation degree of 25%), Da Hong Pao (Fujian, with a fermentation degree of 40%). Black tea: Zhengshan Xiaozhong (Fujian, fully fermented), Ceylon black tea (Fujian, fully fermented).

#### 2.1.2 Reagents

Alum (food grade), sodium chloride, sodium bicarbonate, disodium hydrogen phosphate, potassium chloride, calcium chloride, mucin (all of analytical purity), methanol, acetonitrile, formic acid (all of chromatographic purity), the experimental water is ultrapure water, L-2-chloropropylalanine (chromatographic purity).

### 2.2 Experimental methods

#### 2.2.1 Tea soup preparation

According to GB/T 23776–2023, take 3 g of tea leaves, add 150 mL of 85 °C hot water, cover and steep for 5 min. After filtering through gauze, cool to 25 °C and set aside for later use. After the sample preparation is completed, Maojian green tea is named GT-1, Longjing green tea is named GT-2, Tieguanyin is named OT-1, Da Hong Pao is named OT-2, Ceylon black tea is named BT-1, and Zhengshan Xiaozhongna is named BT-2.

#### 2.2.2 Sensory evaluation

##### 2.2.2.1 Group training

Panelists were recruited from Hunan Agricultural University based on sensory acuity and interest. After training, a 10-member panel was formed. The size of the trained panel (8–12 members) is well-established for dynamic sensory methods like TI and TDS, where intensive training and repeated measures are prioritized over large sample sizes to ensure data precision and panel consistency(Piggott, 2000; Vilela et al., 2026). The study adhered to the Declaration of Helsinki and was approved by the Biomedical Research Ethics Committee of Hunan Agricultural University (No. HNAU-IRB-2024-117). Written informed consent was obtained from all participants.

Panelists underwent 10 sessions focused on astringency intensity assessment. Initially, a 5.0 g/L alum solution served as the reference (intensity score: 10) (Sommer et al., 2022). Training followed standardized TI protocols, using a 10-cm linear scale (0 cm: “none”; 10 cm: “very strong”) with anchors at 1.5 cm (weak; 1% alum), 5.0 cm (medium; 3% alum), and 7.0 cm (strong; 8% alum). Sessions included tasting exercises with tea samples and TI software practice. Proficiency was assessed by curve overlap in repeated TI trials; ≥40% alignment indicated success (Piggott, 2000).

Panelists were also trained in TDS for astringency sub-qualities. Terms were derived from prior studies and refined via discussion to include “harsh,” “puckering,” “coarse grain,” “smooth,” “dry,” and “rough” (usage >60%). Standardized definitions and references were established (Table 1). Training covered TDS software and the “dominant sensation” concept, most noticeable at a given moment, not necessarily strongest. Simulated TDS sessions preceded formal evaluation; performance met predefined standards (Rodrigues et al., 2016).

**Table 1:** Definition and Reference of Astringent sub-quality in TDS evaluation.

**Descriptor**	**Definition**	**Reference**
Harsh	A pungent sensation caused by excessive astringency.	High-concentration edible alum(Gawel, Oberholster and Francis, 2000)
Rough	There is an irregular or raised feeling in the mouth and it is not smooth.	Sandpaper-like texture(Wang et al, 2021b)
Smooth	It has a velvety wrapping feel, smooth and free of rough particles.	Full-fat milk(Zeng et al., 2025)
Puckering	The feeling of contraction and tightening on the surface of the mouth	The astringent sensation caused by eating unripe persimmons(Kang et al., 2019)
Dry	A dry feeling in the mouth caused by lack of lubrication	High-concentration tannin solution(S. Wang et al., 2020)
Coarse grain	Texture related to coarse-grained substances	Solution of grape skin and seed extract(GAWEL et al., 2000)

##### 2.2.2.2 Sample evaluation

**Fig. 1:**
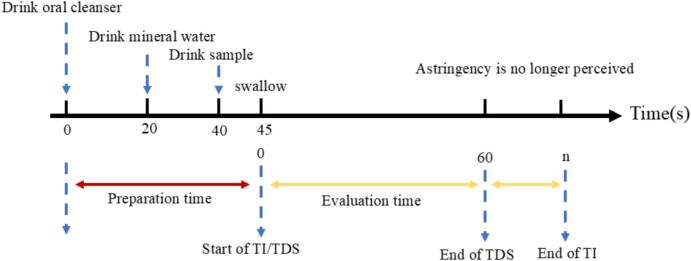
Sensory evaluation process for astringency of TI and TDS.

The experiment was conducted in a controlled sensory laboratory environment. Each tea sample (30 mL) was served in a 50 mL coded ceramic cup and presented to panelists according to a randomized complete block design. To minimize order effects, the presentation sequence was fully randomized across panelists and replicated three times. Standardized tasting protocol (Fig. 1).The evaluation timeline was as follows: 0–20 s, the assessors use oral cleanser; 20–40 s, the assessors rinse their mouths with water and drink 20 mL of the sample, keeping it in their mouths for five seconds without swallowing; At 45 s, the assessors swallow the sample and simultaneously click the software start button to begin the TI or TDS assessment. Between the two tastings, the assessors must wait at least 10 min to reset their taste perception and rinse their mouths with skimmed milk for better oral cleanliness (Lee & Kim, 2013). To eliminate olfactory interference, provide and wear a nose clip throughout the evaluation process. All samples are evaluated in three copies and data is collected using the SensoMaker interface (Pinheiro et al., 2013). The same group simultaneously analyzed the astringency intensity (IS), time intensity (TI) and time advantage perception (TDS) of all tea samples.

For TI analysis, Panelists continuously rated astringency intensity using a computer mouse on a 10-cm linear scale (0 cm = “non-existent”, 10 cm = “very strong”). Intensity data were recorded at 2-s intervals, until the astringency no longer appears. This sampling frequency was chosen because astringency is a persistent oral sensation with relatively slow perceptual changes, and a 2-s interval has been validated in previous TI studies of astringency and bitterness (He et al., 2021). For TDS analysis, the total evaluation duration was fixed at 60 s based on preliminary training trials, which indicated that astringency sub-qualities were no longer dominant beyond this time point. Panelists continuously selected the dominant astringency sub-quality (from six predefined terms: harsh, rough, smooth, puckering, dry, and coarse grain) at each moment. Data were recorded at 1-s intervals. The higher sampling frequency is necessary for TDS because dominant sensations can switch rapidly, and a finer temporal resolution is required to capture these transitions accurately (Pineau et al., 2009; Rodrigues et al., 2016b). To minimize deviation, the sample presentation order was consistent for each panelist across replicates but randomized across panelists at the group level.

#### 2.2.3 Electronic tongue detection of astringency in tea samples

Sample preparation refers to 2.2.1, and the artificial saliva configuration refers to the method of Qi et al. (Qi et al., 2023) Add 5.208 g Sodium bicarbonate,1.369 g Dipotassium phosphate,0.877 g Sodium chloride,0.477 g Potassium Chloride,0.441 g Calcium chloride, and 2.26 g of mucoprotein to 1000 mL of deionized water and adjust the PH value to 7.2. And store it in a refrigerator at −20 °C for later use. Mix the sample with the simulated saliva in a 1:1 ratio. The Electronic Tongue System (TS-5000Z, INSENT, Japan) is applied, which is equipped with six chemical sensor arrays. The reference electrodes include AAE (umami), CT0 (salty), CA0 (sour), C00 (bitter), AE1 (astringent), GL1 (sweet), 30 mM potassium chloride and 0.3 mM tartaric acid. Each tea sample is steeped twice, and each steeping is measured three times. The Weber-Fischer law is a unit of taste, and in this experiment, only the AE1 (astringency) electrode was used.

#### 2.2.4 LC-MS component analysis

##### 2.2.4.1 Sample processing

Accurately weigh 100 ± 5 mg of the sample into a 2 mL centrifuge tube, then add a 6 mm-diameter grinding bead. Next, add 400 μ L of the extraction solvent (methanol: water = 4:1, *v/v*) containing four internal standards (e.g., L-2-chlorophenylalanine at 0.02 mg/mL) to the tube. Grind the mixture using a cryogenic grinder at −10 °C with a frequency of 50 Hz for 6 min. Subsequently, subject the sample to low-temperature ultrasonic extraction (5 °C, 40 kHz) for 30 min. Incubate the sample at −20 °C for 30 min, then centrifuge at 13,000 ×*g* for 15 min (4 °C). Transfer the supernatant to an injection vial with an internal tube for on-machine analysis. Finally, collect 20 μ L of supernatant from each sample, pool them, and prepare quality control (QC) samples.

##### 2.2.4.2 Experimental conditions

The UHPLC-Q Exactive system (Thermo Fisher) was employed for analysis. Chromatographic conditions were as follows: column, ACQUITY UPLC BEH C18 (100 mm × 2.1 mm I. d., 1.7 μ m; Waters, Milford, USA); mobile phase, Phase A (2% acetonitrile in water containing 0.1% formic acid) and Phase B (acetonitrile containing 0.1% formic acid); injection volume, 3 μ L; column temperature, 40 °C; flow rate, 0.4 mL/min; elution gradient: 98% Phase A/2% Phase B at 0 min, maintained until 13 min (5% Phase A), then adjusted to 98% Phase A at 14.5 min, and held through 16 min. Mass spectrometry conditions: ion source, electrospray ionization (ESI); scanning modes, positive and negative ion modes; scan range, 70–1050 *m*/*z*; sheath gas flow rate, 50 arb; auxiliary gas flow rate, 13 arb; ion source heater temperature, 450 °C; capillary temperature, 320 °C; spray voltage, 3500 V (positive) and − 3000 V (negative); S-Lens voltage, 40 V; collision energy, 20%, 40%, and 60%; resolution, 70,000 (Full MS mode) and 17,500 (MS^2^ mode).

### 2.3 Statistical analysis

All data are presented as mean ± standard deviation (SD). For static sensory scores (IS) and electronic tongue data, one-way ANOVA followed by Tukey‘s HSD post hoc test was applied for pairwise comparisons among the six tea samples. For TI and TDS parameters, a three-way mixed-effects model was fitted with sample and evaluator as fixed factors and repetition (three replicates) as a random factor. Post hoc comparisons were performed using Tukey's HSD adjustment for multiple comparisons. A significance threshold of *P* < 0.05 was applied for all tests. The choice of Tukey's HSD (rather than Duncan's test) was made to control the family-wise error rate, as recommended for exploratory studies with multiple pairwise comparisons. Statistical analyses were performed using SPSS version 26.0 (IBM Corp., Armonk, NY, USA).

For TI and TDS data processing, the SensoMaker software was employed to generate TI and TDS curves for each sample. From each TI curve, five parameters were extracted: Iₘₐₓ (maximum intensity observed during evaluation), Tₘₐₓ (time to first reach maximum intensity), Tₜₒₜ (total duration from test start to end), AUC (area under the curve), and 90% Plateau Duration (time intensity remained ≥90% of Iₘₐₓ). For TDS curves, two key parameters were derived: DRₘₐₓ (maximum dominance rate) and Tₘₐₓ (time at which the maximum dominance rate was perceived). The “dominance rate,” defined as the proportion of times a term was cited per instance multiplied by the number of evaluators and repetitions, was calculated to evaluate the dominance of each astringency sub-quality at each time point. TDS graphs plotted both the “ chance level” (baseline dominance rate expected by chance) and “significance level” (minimum ratio threshold for statistical significance). TDS graphs plotted both the “chance level” (baseline dominance rate expected by chance) and “significance level” (minimum ratio threshold for statistical significance). Extracted TI and TDS parameters were analyzed as described above. Sensory trajectories of astringency sub-qualities were further explored using XLSTAT (2021 Edition).

## 3 Results

### 3.1 Intensity scoring

**Fig. 2:**
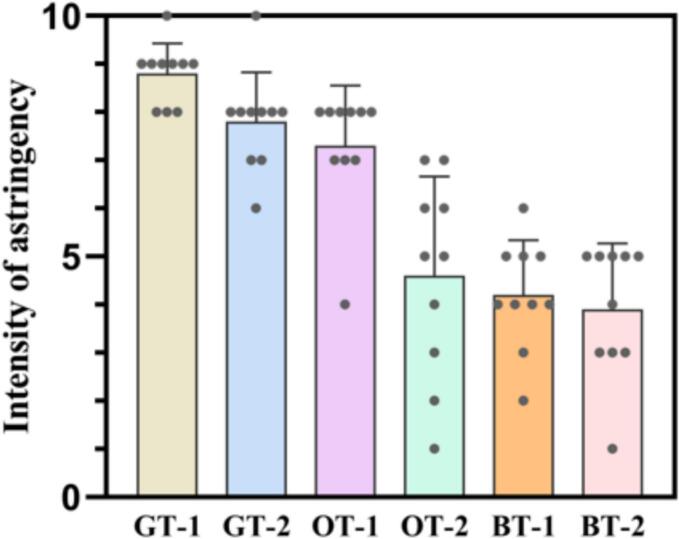
The scoring and evaluation results of the astringency intensity of tea samples. Note: The dots in the figure represent the individual astringency scores of the members of the sensory evaluation panel, and the error bars represent the standard deviations of the astringency scores of the ten members of the sensory evaluation panel.

The static astringency intensity of different tea samples is compared through sensory analysis (IS), and the results are shown in Fig. 2. Statistical analysis shows that there are significant differences in the astringency intensity of different tea samples (*p* < 0.05). As shown by the data distribution, the mean astringency intensity of GT-1 and GT-2 is relatively high with low dispersion, indicating that their astringency is consistently prominent. This observation is consistent with the traditional understanding that green tea usually shows a relatively high intensity of astringency. However, the black tea group (BT-1, BT-2) has a lower intensity of astringency. OT-2 shows moderate astringency, and the data points follow the distinguishable pattern. OT-1 showed a relatively high average astringency intensity, although its data dispersion indicates significant differences between samples. In general, this study clearly proves the decisive impact of tea processing technology on the intensity of astringency. Sensory evaluation results show that the astringency intensity of green tea>oolong tea>black tea shows a consistent trend.

### 3.2 Electronic analysis of tongue astringency in tea samples

Table 2 shows the results of the electronic tongue detection of the astringency of different tea samples. The significance analysis confirmed that the difference in astringency between tea samples was statistically significant (*P* < 0.05). The astringency intensity of green tea samples (GT-1, GT-2) is relatively high, while the astringency intensity of black tea samples (BT-1, BT-2) is relatively low. Oolong tea samples (OT-1, OT-2) showed a moderate astringency level. This is in line with the established trend of decreasing the intensity of astringency as the degree of fermentation deepens, which is consistent with the results of sensory assessment (IS). These findings are consistent with previous studies based on static sensory evaluation (Ye et al., 2022). Specifically, unfermented green tea has the strongest astringency due to its high content of catechins (such as EGCG, ECG) (Košulič et al., 2004; Scharbert et al., 2004); and fully fermented black tea shows the weakest astringency due to the extensive oxidation of catechins. Semi-fermented oolong tea shows a moderate astringency level (Salman et al., 2022). In a word, the change in the intensity of astringency between tea types is fundamentally due to the difference in internal material composition (especially polyphenols) caused by processing(Scharbert et al., 2004; Ye et al., 2022). Further research is needed to clarify the specific component-level differences.

**Table 2:** The intensity of the astringent sensation on the tongue in the tea sample.

	GT-1	GT-2	OT-1	OT-2	BT-1	BT-2
	14.63	12.27	5.27	4.28	3.4	2.73
	14.63	12.57	5.49	4.29	3.54	2.84
	14.63	12.84	5.7	4.36	3.63	2.99
	14.63 ± 0a	12.56 ± 0.23b	5.57 ± 0.18c	4.34 ± 0.04d	3.52 ± 0.09e	2.85 ± 0.11f

The results are presented as mean and standard deviation. Letters a to f indicate significant differences (*P* < 0.05).

### 3.3 Dynamic characteristics of astringency based on TI curves

**Fig. 3:**
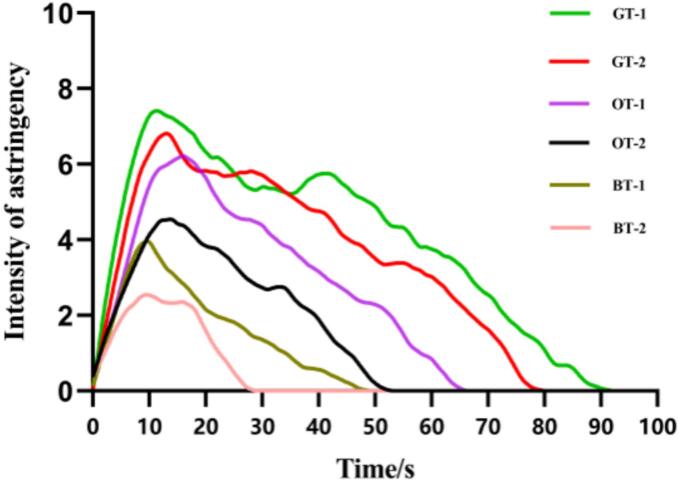
The TI curve of tea samples.

The TI results showed differences in the duration and intensity of astringency between different tea types (Fig. 3). Table 3 presents the results of the analysis of variance of the sample effects in different tea samples and the descriptive statistics of the time-intensity (TI) parameters: maximum intensity (Imax), area under the curve (AUC), time to reach maximum intensity (Tmax), and total duration (Ttot). The data show that the astringency-related TI parameters between different types of teas were significantly different (*P* < 0.05), with green tea samples (GT-1 and GT-2) showing obvious astringency characteristics. The peak intensity (Imax) of GT-1 was 8.27 ± 0.75, its AUC was 405.11 ± 43.73, and its duration (Ttot) was 91.17 ± 8.62 s. For GT-2, the peak intensity was 7.25 ± 0.28 (Imax), AUC was 330.68 ± 36.85, and Ttot was 85.71 ± 10.13 s – both significantly higher than oolong tea and black tea. It is worth noting that GT-1 released its astringency the fastest (Tmax: 9.13 ± 1.04 s), while GT-2 showed the fastest intensity increase (Plateau 90%: 14.01 ± 9.74 s). Conversely, with an increase in the degree of fermentation, the intensity of astringency weakened: black tea (BT-1, BT-2) showed the lowest values for Imax (3.03–4.37), AUC (46.07–95), and Ttot (26.76–37.68 s), and the astringency release speed was slow (BT-2: Tmax 18.01 ± 5.44 s). These results indicate that unfermented green tea exhibits strong and lasting astringency with rapid release, while fermented teas (oolong tea and black tea) show weaker astringency, shorter duration, and slower release kinetics.

**Table 3:** Analysis results of the time-intensity (TI) parameters of the tea sample.

Sample	Imax	Tmax(s)	Auc	Ttot(s)	Plateau 90%(s)
GT-1	8.27 ± 0.75a	9.13 ± 1.04bcd	405.11 ± 43.73a	91.17 ± 8.62a	8.89 ± 4.38b
GT-2	7.25 ± 0.28b	10.65 ± 2.84d	330.68 ± 36.85b	85.71 ± 10.13a	14.01 ± 9.74a
OT-1	6.73 ± 0.38b	12.1 ± 2.43 cd	221.42 ± 28.02bc	62.62 ± 2.66b	7.41 ± 2.87b
OT-2	5.1 ± 0.34c	12.74 ± 1.43bc	147.76 ± 39.43bcd	49.18 ± 3.42c	6.53 ± 2.53b
BT-1	4.37 ± 0.40d	14.59 ± 2.66b	95 ± 25.48 cd	37.68 ± 4.66d	4.5 ± ±1.23b
BT-2	3.03 ± 0.32e	18.01 ± 5.44a	46.07 ± 10.97d	26.76 ± 2.63e	5.26 ± 1.81b
*P* value	0.000	0.000	0.000	0.000	0.003

Different lowercase letters (a–e) within the same row indicate significant differences among tea samples (*P* < 0.05). Imax: maximum intensity observed during evaluation; Tmax: time to first reach maximum intensity; AUC: area under the curve; Ttot: total duration from start to end; Plateau 90%: duration of intensity ≥90% of Imax.

### 3.4 Dynamic dominance analysis of tea astringency sub-quality based on TDS

#### 3.4.1 TDS curve and dominance rate analysis

**Fig. 4:**
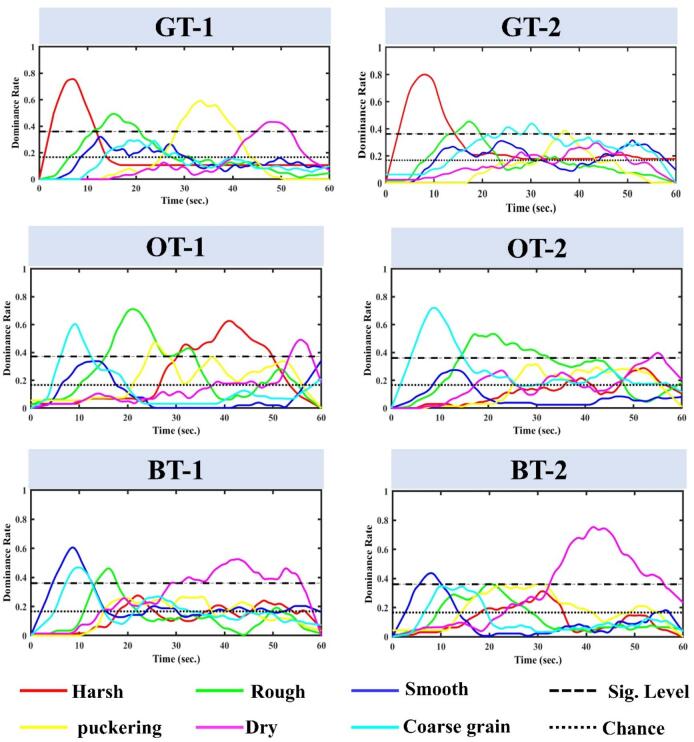
The time advantage perception (TDS) curve of astringency quality in tea samples. The significance level (*p* = 0.05) is indicated by a horizontal dotted line.

The temporal dominance of sensations (TDS) method was used to characterize the dynamic astringency perception in the tea tasting process by identifying the dominant sub-quality perceived at each moment. The time characteristics of astringency were described through sub-qualities such as “harsh,” “rough,” “smooth,” “puckering,” “dry,” and “coarse grain.” The TDS curve (Fig. 4) shows the superior sub-quality of each sample at different periods. By quantifying the maximum dominance rate (DRmax) and peak time (Tmax), dynamic differences in sensory properties of different samples are revealed (Table 4).

In terms of dominance rate (DRmax), tea samples exhibited distinct taxonomic characteristics. OT-1 and OT-2 showed unique characteristics dominated by “rough” and “coarse grain,” with the highest DRmax values for these attributes. Furthermore, in OT-1, “harsh” also demonstrated a significant advantage, revealing its complex layers of astringency. The characteristics of GT-1 and GT-2 were dominated by the “harsh” astringency feature, with a significantly higher intensity of astringency than other tea samples and a strong influence of astringency. In addition, the “dry” strength of BT-2 was exceptionally outstanding (DRmax = 1.02a), significantly higher than that of other tea samples. It formed an astringent taste dominated by “dry” together with BT-1.

In the temporal dimension, the evolution time series (T_max_) of astringency exhibited complex dynamic characteristics as shown in Table 4. The peak of the “harsh” sub-quality for GT-1 and GT-2 appeared earliest, indicating their astringency took effect rapidly and had a strong initial impact. It then successively evolved into “rough,” “coarse grain,” and finally ended with “dry,” forming a complete sensory sequence that transitioned from irritation to physicality and then to convergence. In contrast, the astringency evolution of BT-2 showed a process from “smooth” to “dry,” reflecting the delayed appearance of astringency in black tea. Furthermore, the T_max_ of “coarse grain” varied greatly among different tea samples (from 8.3 s to 36.5 s), suggesting that there may be essential differences in the material basis or mechanism of action that lead to the sensory properties of this sub-quality. In conclusion, the TDS analysis not only distinguished the core astringency characteristics of different tea samples in terms of intensity but also revealed the unique rhythm and sequence of their astringency evolution from the temporal dimension. The differences in the multi-dimensional properties of this astringency are fundamentally caused by the different compositions, degrees of polymerization, and interaction kinetics with oral proteins of polyphenols in each tea sample. This study provides a reliable sensory-physical basis for precisely describing the “quality” and “timing” of tea astringency.

**Table 4:** The maximum dominant rate (DR-max) and occurrence time (T-max) of astringency sub-quality in different tea samples.

Sample	GT-1	GT-2	OT-1	OT-2	BT-1	BT-2	P value
**DRmax**							
Harsh	0.89 ± 0.18a	0.96 ± 0.2a	0.87 ± 0.13a	0.51 ± 0.10b	0.49 ± 0.12b	0.41 ± 0.12b	*P* < 0.001***
Rough	0.71 ± 0.15b	0.61 ± 0.17bc	0.90 ± 0.10a	0.84 ± 0.12b	0.57 ± 0.23bc	0.52 ± 0.22c	*P* < 0.001***
Smooth	0.55 ± 0.15a	0.63 ± 0.29a	0.54 ± 0.25a	0.43 ± 0.19a	0.68 ± 0.18a	0.60 ± 0.27a	0.240NS
Puckering	0.73 ± 0.2a	0.58 ± 0.24a	0.62 ± 0.12a	0.52 ± 0.15a	0.50 ± 0.21a	0.63 ± 0.22a	0.185NS
Dry	0.57 ± 0.13c	0.49 ± 0.18bc	0.61 ± 0.13bc	0.61 ± 0.18bc	0.85 ± 0.21ab	1.02 ± 0.08a	*P* < 0.001***
Coarse grain	0.48 ± 0.07c	0.61 ± 0.27bc	0.82 ± 0.23a	0.78 ± 0.12ab	0.61 ± 0.23bc	0.57 ± 0.23bc	*P* < 0.001***
**Tmax (s)**							
Harsh	6.51 ± 1.81c	6.73 ± 1.36c	39.49 ± 5.63a	41.42 ± 1.41a	44.21 ± 1.36a	25.95 ± 5.44b	*P* < 0.001***
Rough	15.46 ± 1.21b	17.25 ± 1.26b	23.41 ± 5.91ab	29.8 ± 12.10a	22.77 ± 1.46ab	23.19 ± 1.36ab	0.074NS
Smooth	25.31 ± 1.30a	32.55 ± 1.64a	32.79 ± 2.22a	22.71 ± ±1.92ab	12.82 ± 1.48b	8.11 ± 2.28b	0.003**
Puckering	33.10 ± 2.61ab	38.05 ± 3.54a	35.8 ± 9.69ab	38.47 ± 7.80a	25.7 ± 9.65b	30.06 ± 8.89ab	0.068NS
Dry	43.42 ± 1.40ab	40.06 ± 2.31b	51.19 ± 7.9a	47.24 ± 1.27ab	43.64 ± 6.88ab	44.73 ± 6.94ab	0.143NS
Coarse grain	29.56 ± 3.21a	36.49 ± 0.91a	14.69 ± 2.22ab	8.3 ± 2.35c	12.32 ± 6.99c	28.4 ± 2.10bc	*P* < 0.001***

Different lowercase letters (a–c) within the same row indicate significant differences among tea samples (Tukey's HSD, P < 0.05). NS: not significant; *P < 0.05; **P < 0.01; **P < 0.001. DRmax: maximum probability that a sensory attribute becomes dominant; Tmax: time at which dominance reaches its peak.

#### 3.4.2 PCA analysis

**Fig. 5:**
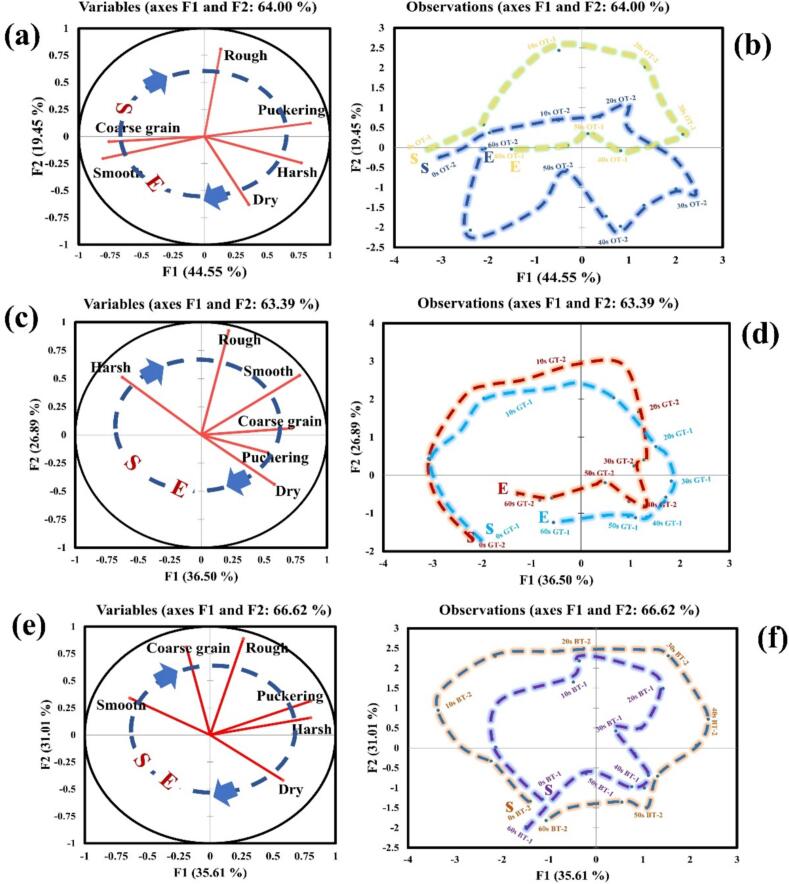
Principal component analysis (PCA) was used to analyze the time advantage of the sensory attributes (TDS) of different tea astringency, and to determine the time sequence of perceived sensory attributes. In TDS, the sensory trajectories of the dominant rates of the six samples are plotted on the PCA biplane map. Figures (a), (c), and (e): They are respectively the sensory trajectory diagrams of the sensory attributes of green tea, oolong tea, and black tea. Figures (b), (d), and (f): Sensory trajectories of green tea, oolong tea, and black tea at different times. Point S represents the starting point of each TDS curve, and point E represents the end point of the TDS curve. (For interpretation of the references to colour in this figure legend, the reader is referred to the web version of this article.)

To visualize the temporal dynamics of astringency sub-qualities across different tea types, principal component analysis (PCA) was performed on the normalized temporal scores of five astringency-related sensory attributes: Harsh, Coarse grain, Puckering, Rough, Smooth, and Dry (Fig. 5). The PCA biplots (Fig. 5a, c, e) illustrate the contribution of each variable to the principal components (F1 and F2), while the score plots (Fig. 5b, d, f) represent the distribution of individual tasting sessions (labeled by tea type, replicate, and time point). For oolong tea (OT), the PCA model explained 64.00% of the total variance (F1: 44.55%, F2: 19.45%). Variables such as “Rough,” “Puckering,” and “Harsh” contributed strongly to F1, indicating that these sub-qualities dominated the early and middle stages of tasting (Fig. 5a). The score plot (Fig. 5b) shows a clear temporal trajectory: samples at 0 s (S) and 60s (E) cluster near the origin, while intermediate time points (10s–50s) spread along the F1 axis, reflecting the dynamic evolution of astringency sub-qualities over time. Similarly, for green tea (GT), the PCA model accounted for 63.39% of the variance (F1: 36.50%, F2: 26.89%). “Rough,” “Smooth,” and “Coarse grain” were the primary contributors to F1, suggesting a distinct sub-quality profile compared to oolong tea (Fig. 5c). The score plot (Fig. 5d) reveals a circular temporal pattern, with samples progressing from “Smooth”-dominant at 0 s to “Rough”-dominant at 60s, highlighting the unique temporal dynamics of green tea astringency. For black tea (BT), the PCA model explained 66.62% of the variance (F1: 35.61%, F2: 31.01%). “Coarse grain,” “Rough,” and “Puckering” were the most influential variables, similar to oolong tea but with a stronger contribution from “Dry” and “Harsh” (Fig. 5e). The score plot (Fig. 5f) shows a compact temporal cluster, with samples at 0 s and 60s forming a closed loop, indicating a more stable and rounded astringency profile over time.

#### 3.4.3 TDS sensing path analysis

To clarify the temporal perception pattern of astringent sub-qualities in tea samples, the sensory trajectory of the dominance rate of six kinds of tea was visualized through PCA biplots within the TDS framework (Fig. 5). These trajectories are divided into three groups: green tea (Fig. 5a and b), oolong tea (Fig. 5c and d), and black tea (Fig. 5e and f), revealing shared temporal structure and obvious differences between the samples. It is worth noting that “dry” became the final main convergent attribute in all samples. However, the initial perception varied depending on the type of tea: green tea began with the dominant position of “harsh,” and as the tasting progressed, it transitioned to “rough,” “smooth,” “coarse grain,” and “puckering” in turn. Oolong tea was mainly “coarse grain,” while black tea began with the perception of “smooth.” Over time, “coarse grain,” “rough,” “puckering,” and “rough” appeared one after another.

The analysis of the perception trajectory diagram (Fig. 5) reveals obvious stage-specific differences in astringency perception between tea samples. It is worth noting that significant changes occurred in the early (0–20 s) and middle (20–40 s) phases of oral processing, but all samples converged to a “dry” dominance in the final stage. Although samples within the same tea category showed roughly similar trajectories, subtle differences between samples still existed – these differences stemmed from the intrinsic characteristics of the samples.

### 3.5 LC-MS analysis

#### 3.5.1 Classification of metabolites

**Fig. 6:**
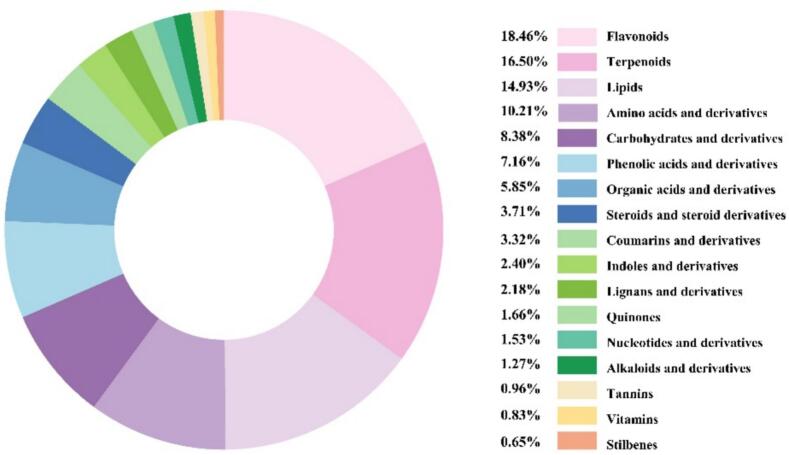
The quantity and classification of metabolites in tea samples. Note: Display the characteristics and percentages of metabolites in each category in descending order. Each different colour in the pie chart in the figure represents a different category, and its area indicates the relative proportion of metabolites in that category.

Tea astringency is closely associated with the types and concentrations of tea polyphenols. To investigate its material basis, we performed LC-MS analysis to examine component differences across teas, identifying a total of 3417 non-volatile compounds (Fig. 6). Detected substances comprised 421 flavonoids, 378 terpenoids, 342 lipids, 234 amino acids, 164 phenolic acids, 134 organic acids, 192 carbohydrates, 164 phenolic acid derivatives, 134 organic acid derivatives, 85 steroids and steroid derivatives, along with quinones, astragalins, and numerous other compounds.

#### 3.5.2 Metabolite cluster analysis

**Fig. 7:**
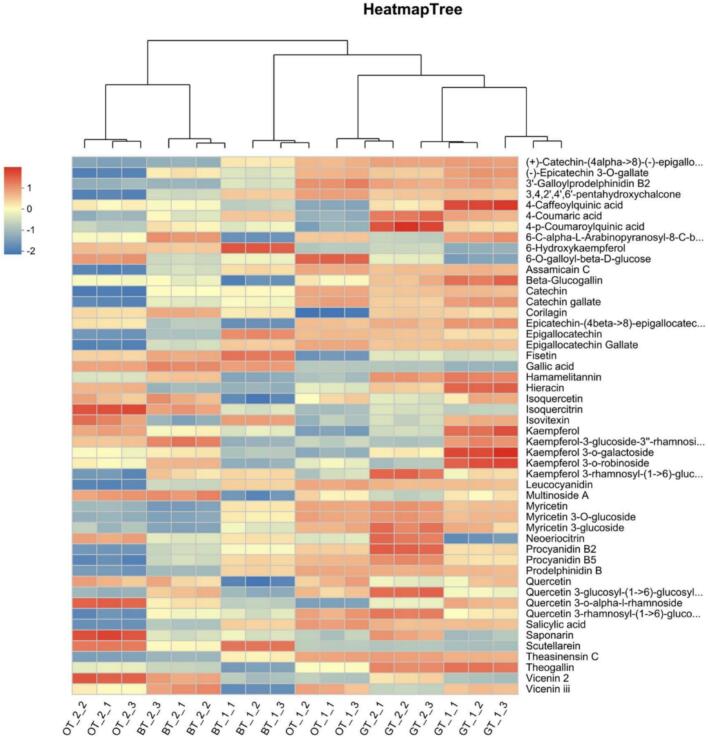
Metabolite cluster map. Note: Each column in the figure represents a sample, and each row represents a metabolite. The colors in the figure indicate the relative expression levels of metabolites in this group of samples, with red representing high expression levels and blue representing low expression levels. (For interpretation of the references to colour in this figure legend, the reader is referred to the web version of this article.)

Heatmap analysis revealed significant clustering of metabolite profiles among tea types (Fig. 7), which correlated strongly with fermentation degree. The relative abundance of characteristic compounds varied markedly across tea types. In green teas (GT-1, GT-2), the mean relative levels of monomeric catechins were the highest, particularly epigallocatechin gallate (7.38) and epigallocatechin (EGC) (7.13). Proanthocyanidins such as procyanidin B2 (7.10) were also highly expressed. These results are consistent with the intense and sharp astringency captured by TI and TDS. In contrast, black teas (BT-1, BT-2) showed a significant reduction in these monomeric and oligomeric polyphenols but exhibited elevated levels of oxidation products, most notably theaflavin digallate (7.12) and theaflavin-3-gallate (6.42). This chemical shift aligns with the delayed and dominant “dry” sensation observed in the TDS analysis of black tea.

Oolong tea samples displayed an intermediate chemical profile. OT-1 retained relatively high levels of EGCG (7.53) and catechin (7.24), correlating with its stronger astringency, while OT-2 had higher levels of oxidized metabolites and L-theanine (6.54), which corresponds to its milder astringency and the unique sensory trajectory revealed by PCA.

GT-1, GT-2, and OT-1 (semi-fermented oolong tea) formed distinct branches in hierarchical clustering due to their relatively high astringency. These teas have distinctive metabolic characteristics. The contents of catechin, epigallocatechin gallate, and proanthocyanidin B2 are significantly higher in these teas compared to black tea.

#### 3.5.3 Correlation analysis of polyphenols - astringency intensity and sub-quality

**Fig. 8:**
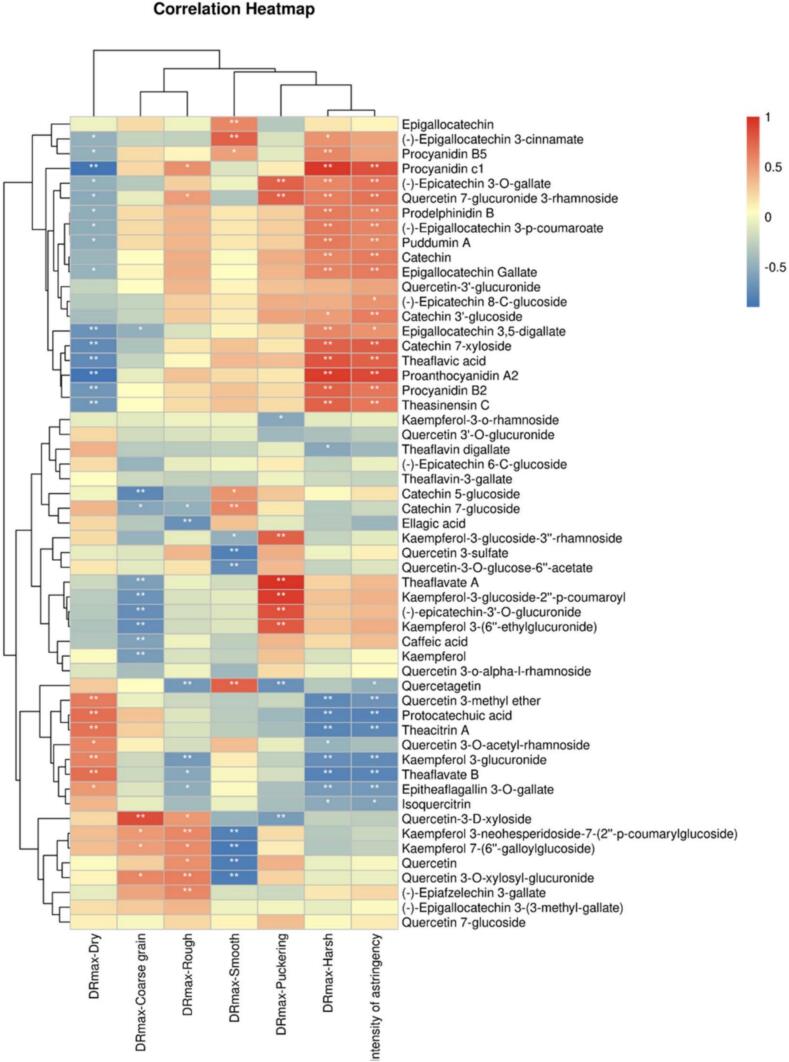
Heat map of the correlation analysis between the dominant rate of the quality and sensory attributes of the main metabolites and the intensity of astringency. Note: Red represents positive correlation, blue represents negative correlation. The depth of the colour indicates the intensity of the correlation. * *p* < 0.05, ** *p* < 0.001. (For interpretation of the references to colour in this figure legend, the reader is referred to the web version of this article.)

This study explored the association mechanism between the composition of polyphenols in tea and the characteristic spectrum of astringency by integrating statistical correlation analysis and sensory evaluation. The results show that the overall intensity of astringency and its sub-characteristics are significantly and specifically associated with different types of polyphenols (Fig. 8). The intensity of astringency is significantly positively correlated with catechins and their oxidized derivatives (such as epigallocatechin gallate (EGCG), epigallocatechin (ECG)), anthocyanins, and quercetin derivatives. This result is consistent with previous studies – condensed tannins and flavonoids/glycosides form insoluble complexes with salivary proteins and are the key material basis for causing the typical astringent sensation (Scharbert et al., 2004). Astringency is not a single sensation but a complex perception composed of multiple tactile sub-characteristics. The features of astringency lie in the coefficient of friction, polyphenol content, sensory analysis, and tannin/salivary protein aggregate characteristics (Brossard et al., 2021; Wang et al., 2024).

Specifically, the “rough” and “coarse grain” sensations showed the strongest positive correlations with highly polymerized catechins (especially EGCG, ECG, and proanthocyanidins) and their derivatives. Their macromolecular structure and high degree of galloylation are hypothesized to enhance binding affinity with salivary proline-rich proteins, potentially leading to the formation of larger, less lubricating aggregates that are perceived as rough or grainy (Hufnagel & Hofmann, 2008). In contrast, the sensation of “dryness” was more strongly associated with oxidized polyphenols, such as theaflavins, and specific phenolic acids (e.g., gallic acid, coumaric acid). It is worth noting that the content of such substances generally increases with the degree of tea fermentation: black tea > oolong tea > green tea. Conversely, the abundant unoxidized catechins (e.g., EGCG, EC) in green tea showed a negative correlation with dryness, which may explain their association with a sharper, more immediate astringency. The “coarse grain” sensation, characterized by a gritty texture, was primarily linked to flavonols (e.g., quercetin and kaempferol glycosides), hesperidin, and quinic acid derivatives. The correlation suggests that these metabolites may contribute to astringency through pathways distinct from those of traditional condensed tannins, although the precise mechanisms (e.g., potential interactions with oral receptors or distinct aggregation behaviors) remain to be elucidated. In summary, these correlative findings reveal distinct associations between specific polyphenol classes and astringency sub-qualities, highlighting how polyphenol structure may regulate the sensory profile of astringency. The data suggest that high-galloylated catechins and proanthocyanidins, capable of forming dense networks via hydrogen bonding with salivary proteins, are strongly linked to “rough” sensations. Meanwhile, fermentation-driven oxidation converts simple catechins into more complex theaflavins, correlating with a shift towards a “drier,” more persistent astringency profile(L. Chen et al., 2021a).

These findings imply that the overall astringency of tea is likely the result of the combined effects of a chemically diverse polyphenol profile. Polyphenols with differing structures may interact with oral proteins and surfaces in varied ways, collectively shaping the complexity of astringency perception. Future research should combine in vitro protein-binding assays, tribological measurements, and targeted sensory studies to move beyond correlation and establish causal molecular mechanisms linking specific polyphenol structures to defined astringency sub-qualities, thereby refining predictive models.

## 4 Discussion

This study, through the combination of multi-dimensional techniques, revealed the dynamic characteristics and material basis of astringency in green tea, black tea and oolong tea. The results not only verified the decisive role of tea processing techniques in flavor quality, but also provided a new theoretical perspective for the regulation of astringency in tea from the sensory-chemical correlation level. The following discussion is carried out from two aspects: the dynamic mechanism of astringency, the sensory-component association, and the methodological value.

### 4.1 Dynamic mechanism of astringency

Previous studies on tea astringency mostly relied on “single sensory scoring” or “static concentration measurement” (such as directly determining the polyphenol content in the tea soup). Although these methods can reflect the “final value” or “total amount” of astringency, they struggle to capture the dynamic process of astringency in the oral cavity from “onset – peak – fading.” This study innovatively adopted a multi-dimensional analysis of TI, TDS, and LC-MS metabolomics to systematically analyze the dynamic expression rules and material basis of astringency in green tea, oolong tea, and black tea.

The study systematically revealed the dynamic characteristics of astringency in green tea. The maximum astringency intensity (Imax) of typical green teas (such as GT-1 and GT-2) was significantly higher than that of oolong tea and black tea, confirming its stronger overall astringency. Meanwhile, the peak time (Tmax) of green tea was significantly shorter, much lower than that of oolong tea (21 to 25 s) and black tea, indicating that its astringency has a rapid onset characteristic. This difference may be related to the degree of fermentation. Although no research has directly discussed this for tea, a study on the pungency of Baijiu also observed a similar dynamic difference, where aging time significantly affected the intensity and duration of pungency (He et al., 2021b). The PCA sensory trajectory maps (Fig. 5) provide a novel visualization of how astringency sub-qualities evolve over time in a sample-specific manner. The trajectories reveal that all six tea samples converge to ‘dry’ as the terminal dominant attribute, suggesting that oral moisture loss represents the final common pathway of astringency perception regardless of tea type. However, the initial and intermediate perceptual pathways diverge markedly. Green teas (GT-1 and GT-2) originate in the ‘harsh’ quadrant and traverse through ‘rough’ and ‘coarse grain’ before reaching ‘dry,’ reflecting a sequence from rapid protein precipitation (harsh) to particulate sensation (coarse grain) and finally mucosal dehydration. Oolong teas, particularly OT-2, show a trajectory dominated by ‘coarse grain’ and ‘rough’ with minimal ‘harsh’ expression, consistent with their intermediate oxidation status and the presence of medium-molecular-weight polyphenol aggregates. Black teas initiate with ‘smooth’—a unique finding—before transitioning to ‘dry,’ which may reflect the initial lubricating effect of theanine and other non-astringent components prior to the delayed interaction of theaflavins with salivary mucins. These time-resolved perceptual pathways provide a new framework for understanding how tea processing chemistry shapes the temporal sequence of mouthfeel sensations.

Furthermore, LC-MS analysis indicated that an increase in the content of astringent substances significantly prolonged the duration and intensity of astringency. This is consistent with a study on the influence of the pungent taste of pepper oil resin on the concentration and composition of alkylamines, which found that a higher concentration of alkylamines significantly enhanced the overall intensity of irritation, dynamic characteristics, and sub-quality duration (Song et al., 2025). TDS reveals the differences in the sub-qualities of astringency among different types of tea through the real-time dominance rate (DR) of six astringency sub-attributes (Harsh, Rough, Smooth, Puckering, Dry, and Coarse grain). Green tea, with “harsh sensation” and “coarse grain sensation” (secondary dominance in the middle stage) as its core, reflects the rapid harsh effect of high content of free catechins. Oolong tea is characterized by an alternating dominance of “rough” and “coarse grain,” which is related to the uneven oxidation of polyphenols caused by semi-fermentation. Black tea is characterized by a “dry” sensation, accompanied by a brief “smooth” at the beginning. It is reported that different astringent substances can produce different astringent sub-qualities. For instance, catechins have been proven to cause the astringent property of “rough,” while flavanol glycosides cause the property of “smooth” (Liu & Tzen, 2022). The astringency quality of different tea samples varies significantly, which is related to the composition and content of polyphenols. In addition, the analysis found that the intensity of astringency is closely related to the composition of the sub-quality. For instance, a high-intensity astringency has a significant connection with “harshness.” Future research can further analyze the relationship between the intensity of astringency and the sub-quality of astringency.

### 4.2 Astringency-related ingredients

The perception of astringency arises from multifaceted physical and chemical interactions in the oral cavity. Research indicates that different astringency sub-qualities may be driven by distinct mechanisms (Wang et al., 2021). For instance, dryness and roughness are driven by the adsorption of tannins and salivary films, which can lead to poor lubricity, film peeling, and thus exhibit high boundary friction in tribology. In contrast, the sensation of puckering may be linked to alterations in the viscoelastic properties of the salivary film, manifesting as a rapid rise in friction. This study builds upon this mechanistic framework by investigating how variations in polyphenol composition correlate with specific astringency sub-qualities.

LC-MS metabolomics analysis revealed the material basis of the dynamic differences in astringency. The analysis confirmed that catechins (e.g., EGCG, ECG), their oxidation products, anthocyanins, and quercetin derivatives are key compounds associated with astringency, consistent with prior literature (Y.-H. Chen et al., 2022; Wu et al., 2023; Ye et al., 2022b). In our study, these compounds showed a strong positive correlation with the TI parameter for maximum intensity (Imax). More specifically, TDS analysis revealed a significant association between these compounds and the perception of “harshness.” This finding aligns with the established linear relationship between catechin concentration (notably EGCG and ECG) and overall astringency intensity (Xu et al., 2018). Green tea, unfermented, retains approximately 85% of its tea polyphenols, among which epigallocatechin gallate (EGCG) is the main contributor to the astringency (Scharbert et al., 2004). During the processing of oolong tea (semi-fermented) and black tea (fully fermented), enzymatic oxidation polymerizes these polyphenols into compounds like theaflavins and thearubigins. These larger molecular weight compounds are generally associated with a milder astringency. (Wen et al., 2023). While previous research established the link between these compounds and astringency, the time-resolved data from TI in this study further delineated the temporal characteristics. The astringency in green tea, driven by catechins like EGCG, was characterized by a rapid onset and a pronounced “harsh” peak. This rapid perception could be related to the molecular structure of these catechins, which facilitates prompt interaction with salivary proteins (e.g., proline-rich proteins) through hydrogen bonding and hydrophobic effects. In black tea, LC-MS detected a significant enrichment of the characteristic component theaflavin-3′-gallate (TF3’G). TDS analysis showed that the perception associated with TF3’G evolved differently, being strongly linked to the “dry” sub-quality, which dominated the middle to later stages of the evaluation. This presents a contrast to the immediate “harshness” of green tea, suggesting a slower-developing, more persistent astringent character. The delayed onset and sustained nature of this “dryness” may be related to the larger molecular size and more complex structure of TF3’G, which could affect its diffusion and interaction kinetics with oral surfaces, though the precise mechanisms warrant further investigation. This contrast between the rapid “harshness” of green tea catechins and the delayed “dryness” associated with black tea theaflavins underscores the diversity of astringency sub-qualities. It is also noteworthy that a high level of theanine was detected in sample OT-1. Theanine is known for its taste-masking or moderating effects (Hao et al., 2024; Sun et al., 2023), which could explain why OT-1 exhibited lower TI parameters than typical green teas but higher than black teas.

The results of electronic tongue detection showed a high degree of consistency with the overall sensory evaluation of astringency intensity, objectively verifying the objective existence of differences in astringency intensity among different types of tea. However, the electronic tongue has obvious limitations in distinguishing the attributes of the astringent sub-type. For sample OT-2, the electronic tongue indicated an intermediate astringency intensity between green and black tea but provided no information on sub-quality. TDS analysis precisely captured that OT-2's astringency was dominated by a “rough” sensation between 10 and 30 s. Based on the LC-MS data and literature, this specific “rough” sub-quality may be associated with the presence of high-molecular-weight proanthocyanidins in OT-2. Such polymeric polyphenols are known to form less soluble aggregates with salivary proteins, which could contribute to a coarse, grating tactile sensation perceived as roughness (Vidal et al., 2004).

## 5 Conclusion

This study integrated TI, TDS, electronic tongue, and LC-MS to analyze tea astringency. Results showed an intensity ranking: green tea > oolong tea > black tea. Green tea exhibited the highest astringency parameters (Imax, AUC, Ttot) with the fastest onset (shortest Tmax), while black tea showed the lowest values and slowest release. TDS revealed that green tea was dominated by “harsh” and “coarse grain” sensations, oolong tea by alternating “rough” and “coarse grain,” and black tea by a late “dry” sensation. Chemically, green tea's EGCG correlated with “harshness,” black tea's theaflavins with “dryness,” and oolong tea's proanthocyanidins B5/C1 with “coarse grain” and “roughness,” their polymers forming complex astringent layers. These findings clarified astringency dynamics and its material bases for flavor regulation in tea.

## CRediT authorship contribution statement

**Wei Mao:** Writing – review & editing, Writing – original draft, Methodology, Investigation, Formal analysis, Data curation. **Yajuan Li:** Writing – review & editing. **Fangfang Yan:** Writing – review & editing. **Hui Zhou:** Writing – review & editing. **Xiankang Fan:** Writing – review & editing. **Zhonghua Liu:** Writing – review & editing. **Maiquan Li:** Writing – review & editing, Conceptualization. **Jie Luo:** Writing – review & editing, Project administration, Funding acquisition, Conceptualization.

## Declaration of competing interest

The authors declare that they have no known competing financial interests or personal relationships that could have appeared to influence the work reported in this paper.

## Data Availability

Data will be made available on request.
